# DHX37 variants in patients with 46,XY disorders or differences of sex development

**DOI:** 10.1038/s41439-025-00322-2

**Published:** 2025-09-08

**Authors:** Yuko Katoh-Fukui, Daisuke Saito, Hiroko Narumi, Atsushi Hattori, Maki Igarashi, Erika Uehara, Hirohito Shima, Junko Kanno, Yukihiro Hasegawa, Reiko Horikawa, Keisuke Nagasaki, Maki Fukami

**Affiliations:** 1 Department of Molecular Endocrinology, National Research Institute for Child Health and Development, Tokyo, Japan; 2 Division of Endocrinology and Metabolism, Tokyo Metropolitan Children’s Medical Center, Tokyo, Japan; 3 Division of Endocrinology and Metabolism, National Center for Child Health and Development, Tokyo, Japan; 4 Department of Pediatrics, Tohoku University School of Medicine, Sendai, Japan; 5 Division of Pediatrics, Department of Homeostatic Regulation and Development, Niigata University Graduate School of Medical and Dental Sciences, Niigata, Japan

**Keywords:** Genetic testing, Gonadal disorders

## Abstract

Here, using whole-exome sequencing of a cohort of 17 Japanese patients with 46,XY disorders or differences of sex development, we identified two pathogenic DEAH-box helicase 37 (DHX37) variants in three patients. We also identified a patient with a likely pathogenic variant in *SOX9* and a rare likely benign variant in DHX37. This Data Report highlights the genetic and phenotypic diversity of *DXH37* variants.

## Data report

DEAH-box helicase 37 (DHX37), a member of the RNA helicase family, plays essential roles in ribosome biogenesis and splicing^1^. More than 50 patients with deleterious DHX37 missense variants have been reported^2^. Most of the reported variants, located in the phylogenetically conserved ATP- and RNA-binding domains^1^, have been detected in patients with testicular regression syndrome (TRS) or partial gonadal dysgenesis (PGD), which are types of nonsyndromic 46,XY disorders or differences of sex development (DSD). These reports primarily focused on DHX37 missense variants as a monogenic cause of 46,XY DSD^3–6^. Moreover the co-occurrence of rare DHX37 and other gene variants has been noted^7–10^, although the number of cases with TRS or PGD remains limited^7,9^. This study aimed to address the genetic and phenotypic diversity of DHX37 variants in patients with 46,XY DSD.

Whole-exome sequencing was conducted on 17 Japanese patients with nonsyndromic 46,XY DSD associated with dysgenic gonads and atypical external genitalia (Supplementary Methods). The presence of rare variants, including missense, nonsense, gain/loss of stop codons, frameshift and splicing variants, with allele frequencies of <1% was queried in the following databases: 1000 Genome Browser (http://www.ncbi.nlm.nih.gov/variation/tools/1000genomes/), jMorp (Tohoku Medical Megabank Organization Japanese Multi Omics Reference Panel; https://jmorp.megabank.tohoku.ac.jp/), dbSNP (http://www.ncbi.nlm.nih.gov/snp/) and gnomAD (https://gnomad.broadinstitute.org). The functional consequences of substitutions were predicted using Combined Annotation Dependent Depletion (CADD; http://cadd.gs.washington.edu), with scores ≥20 considered damaging. Variants were examined in 65 genes associated with the etiology of 46,XY DSD^11,12^ (Supplementary Methods). Three patients (patients 1–3) harbored previously reported pathogenic DHX37 variants (NM_032656; c.1000C>T; p.(Arg334Trp) and c.2020C>T;p.(Arg674Trp)) (Table 1). Moreover, in a previously reported case, patient 4, who had a heterozygous missense substitution in *SOX9* (NM_000346.3; c.1309C>T; p.(Arg437Cys))^13^ classified as likely pathogenic, harbored a rare DHX37 variant (NM_032656; c.1283C>T; p.(Pro428Leu)) classified as likely benign, which has not been previously reported in patients with 46,XY DSD (Table 1 and Supplementary Table 1). In the surveillance of the rare variants in patients 1–4, any other deleterious variants in 65 genes associated with 46,XY DSD were not identified.

**Table 1:** Summary of the variants.

Patient no.	Phenotype	Gene	Variant	SNP ID	Zygosity	Allele frequencies			In silico prediction		ACMG guidelines^b^	
						gnomAD v4.4.0	Highest-frequency of gnomAD	ToMMo 14KJPN	GADD_phrd	REVEL	Classification	Evaluation criteria
1	TRS	DHX37	p.Arg334Trp	ND	Hetero	ND	ND	ND	35 3	0.595	Pathogenic	PS1 + PM1 + PM2 + PP3 + PP4
2	TRS	DHX37	p.Arg334Trp	ND	Hetero	ND	ND	ND	35 3	0.595	Pathogenic	PS1 + PM1 + PM2 + PP3 + PP4
3	PGD	DHX37	p.Arg674Trp	rs1954336272	Hetero	ND	ND	ND	33 3	0.913	Pathogenic	PS1 + PM1 + PM2 + PP3 + PP4
4^a^	TRS	DHX37	p.Pro428Leu	rs766672697	Hetero	0.00002	0.0002228 (EastAsian)	0.0005	23.3	0.351	Likely benign	PP4 + BS1 + BP4
		*SOX9*	p.Arg437Cys	rs1189441865	Hetero	0.000009	0.00002228 (EastAsian)	0.00004	28.8	0.772	Likely pathogenic	PS3 + PM2 + PP3 + PP4

^a^The patient has been reported previously with SOX9 variant c.1309C>T (p.Arg437Cys)^13^.

^b^ACMG guideline^18^ PS1: same amino acid change as a previously established pathogenic variant regardless of nucleotide change. PS3: well-established in vitro or in vivo functional studies supportive of a damaging effect on the gene or gene product. PM1: located in a mutational hot spot and/or critical and well-established functional domain without benign variation. PM2: absent from controls (or at extremely low frequency if recessive) in Exome Sequencing Project. PP3: multiple lines of computational evidence support a deleterious effect on the gene or gene product (conservation, evolutionary, splicing impact and so on). PP4: patient’s phenotype or family history is highly specific for a disease with a single genetic etiology BS1: allele frequency is greater than expected for disorder. BP4: multiple lines of computational evidence suggest no impact on gene or gene product. Pathogenic: 1 strong (PS1–PS4), and 2 moderate (PM1–PM6) and ≥2 supporting (PP1–PP5). Likely pathogenic: 1 strong (PS1–PS4) and 1–2 moderate (PM1–PM6), or 1 strong (PS1–PS4) and ≥2 supporting (PP1–PP5). Likely benign: 1 strong (BS1–BS4) and 1 supporting (BP1–BP7).

ND, no data.

The clinical information of the four patients with rare DHX37 variants is summarized in Table 2. The external genitalia of the patients were evaluated using the Quigley scale and the external masculinization score (EMS) (range 0–12)^14^, and the masculinization defects were categorized according to the clinical 46,XY DSD categories^15,16^ as follows: male external genitalia with a streak or rudimentary gonad and the disappearance of Müllerian derivatives (46,XY TRS); ambiguous genitalia with a streak or rudimentary gonad and the disappearance of Müllerian derivatives (46,XY PGD); and complete female-type external genitalia with a streak or rudimentary gonad and Müllerian derivatives (46,XY CGD) (Table 2). Patient 1 had a buried phallus and masculine external genitalia. The inguinal gonads, which were palpable at 2 years of age, were not palpable at 6 years and 2 months of age, with only rudimentary gonads detected via echography. The Tanner stages for genitalia and pubic hair were 1 and 3, respectively, at the age of 33 years and 10 months. Patient 2 had a small phallus with mild hypospadias but was raised as a female. Histopathologic assessment revealed gonadal stroma-like tissue, with gonad volumes of 0.72 and 0.66 ml for the right and left gonads, respectively (reference volume, approximately 2 ml). Patient 3 had a phallus with severe perineal hypospadias and was raised as a female. The right and left gonadal volumes were 0.48 and 0.53 ml (reference volume: approximately 2 ml), respectively. Histopathologic evaluation did not detect seminiferous tubules in the rudimentary testes. Patient 4 had been reported previously by our group^13^. The patient had a borderline micropenis with mild hypospadias. The bilateral testes were not palpable at birth. At the age of 2 years and 6 months, laparoscopic evaluation identified fibrous tissue without germ cells in the bilateral inguinal canals. The phenotypes common to all four patients included the absence or residual presence of Müllerian structures and hypogonadism.

**Table 2:** Clinical characteristics of the four 46,XY DSD patients with variants in DSD-related genes.

Patient no.	Diagnosis	Variants in DSD-related genes (heterozygous)	Sex^a^	Quigley scale^b^	EMS^c^	Length of the phallus	Testicular palpation	Urethral meatus	Scrotum/labia	Vagina	Gonads	Epididymis	Uterus	Fallopian tubes/ oviduct	Treatment	Age at hormone testing	LH level (mIU/ml)		FSH level (mIU/ml)		T level (ng/dl)	
																	Basal	Peak^d^	Basal	Peak^d^	Basal	Peak^e^
1	TRS	DHX37 Arg334Trp	Male	3	6 > 4	Buried (2 y 1 m), 1.5 × 2 cm (33 y 10 m)	Palpable (inguinal) (2 y 1 m), not palpable (6 y 2 m, 26 y 5 m, 33 y 10 m)	Penile (26 y 5 m)	Scrotum (immature) (26 y 5 m)	Present (not open) (6 y 2 m, 26 y 5 m)	LR: atrophic testes, no response to hCG stimulation (6 y 2 m); LR: rudimentary testes (26 y 5 m); LR: rudimentary gonad (33 y 10 m)	Present (immature) (26 y 5 m)	Absent (6 y 2 m, 26 y 5 m)	Absent (33 y 10 m)	Bilateral testicular fixation (6 y 2 m), testosterone enanthate (33 y 11 m)	26 y 5 m 33 y 11 m 34 y 6 m	13.6 [0.8–5.7] 8.8 [0.8–5.7] 13.3 [0.8–5.7]	ND ND ND	54.4 [2.0–8.3] 29.0 [2.0–8.3] 33.6 [2.0–8.3]	ND ND ND	15.1 [131–871] ≤0.03 [131–871] 0.7 [131–871]	ND ND ND
2	TRS	DHX37 Arg334Trp	Female	1	7	0.9 cm (d 21)	Not palpable (d 21)	Apex (1 y 1 m)	Scrotum (1 y 1 m)	Present (laparoscopy) (d 21)	LR: streak; R: ovarian stroma-like (1 y 1 m)	Present (1 y 1 m)	Absent (1 y 1 m)	Present (1 y 1 m)	Gonadectomy (1 y 1 m), vulvoplasty (1 y 8 m)	21 d 9 y	0.7 24.6 [0.0–0.4]	12.2 154.5 [0.4–6.0]	26.5 111.6 [0.6–3.0]	189.5 150 [6.3–15.6]	0,2 ≤0.03 [14–22]	0.2 ND
3	PGD	DHX37 Arg674Trp	Female	5	1	2.7 cm (5 y 11 m)	Not palpable (5 y 11 m)	Perineal (5 y 11 m)	Labia (5 y 11 m)	Present (5 y 11 m)	LR: near the inguinal canal, rudimentary testes (5 y 11 m); LR: no seminiferous tubules (6 y 10 m)	Present (5 y 11 m)	Present (residual) (5 y 11 m)	Present (6 y 10 m)	Gonadectomy, vulvoplasty (6 y 10 m), estrogen (12 y 2 m)	5 y 11 m 8 y 10 m	1.5 [0.0–0.4] 17.9 [0.0–0.4]	35.7 [0.4–6.0] ND	43.0 [0.6–3.0] 65.3 [0.6–3.0]	116.8 [6.3–15.6] ND	4.0 [14–22] 12.7 [27–39]	4.0 [146–200] ND
4	TRS	*SOX9* Arg437Cys^f^ DHX37 Pro428Leu	Male	2	6	2.5 cm (2 y 9 m)	Not palpable (at birth)	Apex (at birth)	Scrotum (at birth)	ND	LR: fibrous tissues (2 y 9 m)	ND	Absent (2 y 9 m)	ND	ND	2 y 8 m	2.8 [<0.3–1.3]		109.8 [0.4–1.5]		<3 [6–16]	<3 [>20]

Numbers in parenthesis indicate the age at testing in years (y), months (m) and days (d).

L, left; R, right; LH, luteinizing hormone; FSH, follicle-stimulating hormone; T, testosterone; ND, not done.

The conversion factors to the SI unit are LH level, 1.0 IU/l; FSH level, 1.0 IU/l; and testosterone level, 0.03467 nmol/l. The numbers in square brackets indicate the reference ranges for males.

^a^Sex, assigned sex, ^b^PMID: 7671849 DOI: 10.1210/edrv-16-3-271, ^c^PMID: 10619959 DOI: 10.1046/j.1464-410x.2000.00354.x, ^d^luteinizing hormone-releasing hormone-stimulated, ^e^human chorionic gonadotropin-stimulated.

^f^The patient had *SOX9* variant c.1309C>T (p.Arg437Cys) as described in a previous study^13^.

A notable clinical feature is the disappearance of palpable testes during childhood in patient 1. In most previously reported neonatal and infant patients with DHX37 variants, testicular regression occurred before birth, presumably during the masculinization process. However, patient 1 exhibited progressive postnatal gonadal disappearance. The gradual disease progression observed in this patient suggests that DHX37 plays a crucial role in testis maintenance during childhood. Patients with pathogenic DHX37 variants may require personalized assessment with long-term follow-up.

The pathogenic variants associated with TRS and PGD observed in the current cohort and reported in previous studies (Supplementary Table 1) indicate that pathogenic DHX37 variants predominantly occur in a specific number of amino acid residues. Recently, de Oliveira et al. classified DHX37 variants into pathogenic and other groups based on the conservation of amino acid residues in which mutations occur^1^. Two DHX37 variants detected in patient 1–3 were consistently located in the RNA- or ATP-binding motifs within the RecA1/2 domain^6^. A high pathogenicity score based on in silico prediction with the CADD score (>25), a low minor allele frequency (<0.0001) in the general population, and the location of the variants in conserved motifs within the RecA1/2 domain are crucial in classifying these variants as contributors to 46,XY DSD in three of the four patients with pathogenic *D*HX37 variants in this study. Although the DHX37 variant p.(Pro428Leu) has not been previously reported in patients with 46,XY DSD, it is located outside the phylogenetically conserved motifs in the RecA2 domains. The maximum allele frequency of the DHX37 variant p.(Pro428Leu) range was 0.0002228 (East Asia, gnomAD), which is a magnitude higher than other DHX37 pathogenic variants (Table 1). Moreover, the rare exome variant ensemble learner (REVEL) score (https://sites.google.com/site/revelgenomics/) was relatively low^17^ (Table 1).

This study reports the co-occurrence of rare *SOX9* and DHX37 variants in a patient with 46,XY DSD. *SOX9* plays a critical role in testicular determination and the maintenance of *SOX9* expression in Sertoli cells^18^. The *SOX9* variant p.(Arg437Cys), which was observed in patient 4, involves a highly conserved amino acid residue, with a reported allele frequency ranging from 0.000009 (gnomAD v4.1.0) to 0.000033 (ToMMo 60KJPN) (Table 1). The *SOX9* variant p.(Arg437Cys) retains normal in vitro activity for the transactivation of the *Col2a1* enhancer but exerts impaired activity for the *Amh* promoter^13^. Meanwhile, DHX37 is expressed in presupporting and supporting Sertoli cells in the human fetus^4^. According to the American College of Medical Genetics and Genomics guidelines for the interpretation of sequence variants^19^, the *SOX9* p.(Arg437Cys) and DHX37 p.(Pro428Leu) variants were assessed as likely pathogenic and likely benign, respectively (Table 1). These results indicate that the *SOX9* variant in patient 4 is a monogenic cause of 46,XY DSD. However, it remains possible that the DHX37 variant modified the phenotype of patient 4. In this regard, six known cases of 46,XY DSD with rare variants in DHX37 and other 46,XY DSD-causative genes have been reported^7–10^. These patients exhibited diverse phenotypes ranging from female- to male-type external genitalia (Supplementary Table 2). The gonads of the cases also varied, with some showing palpable testes and others presenting with streak gonads. Notably, a patient with a DHX37 pathogenic variant and a *MAMLD1* rare variant and two patients with *NR5A1* frameshift variants and a DHX37 VUS/LB variant had no uterus, with no evidence of the presence of testes or attenuation of testosterone (Supplementary Table 2). Certainly, in these reported patients with *NR5A1* frameshift variants, the phenotype can be explained entirely by the *NR5A1* variants. Nevertheless, the effects of the *SOX9* and DHX37 variants on the phenotype of patient 4 remain uncertain, as there are no notable phenotypic differences between patients 1–3 and patient 4.

In conclusion, whole-exome sequencing was carried out on 17 patients with nonsyndromic 46,XY DSD, and results identified two pathogenic DHX37 variants in three patients. In addition, rare DHX37 and *SOX9* variants were detected in a patient with dysgenic gonads. Four patients presented with 46,XY DSD of various severities. The study results highlight the genetic and phenotypic diversity of pathogenic DHX37 variants, in addition to the importance of DHX37 variants as a cause of 46,XY DSD.

## Supplementary information

Supplementary Methods

## Data Availability

Some datasets generated and/or analyzed in the current study are available from the corresponding author upon reasonable request.
