# Supplementary material for: DHX37 variants in patients with 46,XY disorders or differences of sex development
Source: Hum Genome Var. 2025 Sep 8;12:18. doi: 10.1038/s41439-025-00322-2 (PMC12415111; doi:10.1038/s41439-025-00322-2)
Supplement: Supplementary file 1 — Supplementary Methods [file 41439_2025_322_MOESM1_ESM.docx]

**Supplemental Materials for**

***DHX37* variants in patients with 46,XY disorders/differences of sex development**

Yuko Katoh-Fukui^＊^, Daisuke Saito, Hiroko Narumi, Atsushi Hattori, Maki Igarashi, Erika Uehara, Hirohito Shima, Junko Kanno, Yukihiro Hasegawa, Reiko Horikawa, Keisuke Nagasaki, and Maki Fukami

^＊^Correspondence: Yuko Katoh-Fukui

Email: fukui-y@ncchd.go.jp

**This PDF file includes:**

Supplementary Methods

Table S1

Table S2

# Supplemental Methods

# Whole-exome sequencing

A library was constructed using the SureSelect Human All Exon kit (V6, Agilent Technologies, Santa Clara, CA, USA) and sequenced as 150-bp paired-end reads on a NovaSeq 6000 sequencer (Illumina, San Diego, CA, USA). Base calling, read filtering, and demultiplexing were performed using the standard Illumina pipeline. Sequence reads were mapped to the human reference genome (hg19/GRCh37.p13) using BWA 0.7.17 (https://sourceforge.net/projects/bio-bwa/files). Local realignment, quality score recalibration, and variant calling were performed with GATK3.8 (https://anaconda.org/bioconda/gatk). The called variants were annotated by ANNOVAR (http://www.openbioinformatics.org/annovar/). Variants were referred to the 65 genes associated with 46,XY,DSD etiology; *AKR1C2*, *AKR1C4*, *AMH*, *AMHR2*, *AR*, *ARX*, *ATF3*, *ATRX*, *BBS9*, *CBX2.1*, *CDKN1C*, *CHD7*, *CYB5A*, *CYP11A1*, *CYP17A1*, *CYP19A1*, *DHH*, *DHX37*, *DMRT1*, *DMRT2*, *ESR2*, *FGF8*, *FGFR1*, *FGFR2*, *FSHB*, *GATA4*, *GNRH1*, *GNRHR*, *HESX1*, *HHAT*, *HMGCS2*, *HOXA13*, *HSD17B3*, *HSD3B2*, *INSL3*, *KAL1*, *KISS1R*. *LEP*, *LHCGR*, *LHX3*, *LHX9*, *MAMLD1*, *MAP3K1*, *MYRF*, *NR0B1*, *NR5A1*, *POR*, *PPP2R3C*, *PROK2*, *PROKR2*, *PROP1*, *RXFP2*, *SOX8*, *SOX9*, *SRD5A2*, *SRY*, *STAR*, *STARD8*, *TAC3*, *TSPYL1*, *WDR11*, *WNT4*, *WT1*, *WWOX*, *ZFPM2*

**Supplemental Tables**

| **Table S1. Reported cases of patients with rare *DHX37* variants, including those presented in this report.** | | | | | | | | | | | | | | | | | | | | | | |
| --- | --- | --- | --- | --- | --- | --- | --- | --- | --- | --- | --- | --- | --- | --- | --- | --- | --- | --- | --- | --- | --- | --- |
| DSD subtype |  | *DHX37* rare variants^†^ | | | | | | | | | | | | | | | | | | | | |
| Amino acid |  | Ile254 | Gly302 | Thr304 | Arg308 | Arg334 | Arg390 | Ser408 | Pro428 | Leu467 | Thr477 | Gly478 | Arg487 | Ala492 | Asp506 | Ser595 | Ser626 | Leu627 | Arg674 | Ala737 | Val999 | Gly1030 |
| Motif^‡^ |  | – | – | Ia | Ia | Ib | – | III | – | – | IV | IV | – | – | – | IVa | V + Va | V + Va | VI | – | – | – |
| 46,XY CGD |  | – |  | – | – | – | His | – | – | – | – | – | – | – | – | – | – | – | – | – | – | – |
| 46,XY PGD^§^ |  | – | Ser^¶^ | Met (2) | Gln (7) | Leu | – | Leu | – | Val^¶^ | Met | – | – | – | – | Phe | – | Phe | **Trp** (3) Gln (3) | – | Met^¶^(2) |  |
| 46,XY TRS^#^ |  | – |  | – | Gln(10) | **Trp** (4) | – | Leu | **Leu**^¶^ | – | Met^††^ | Arg | – | – | – | Phe | Leu | – | Trp (5) | – | – | Glu |
| Other 46,XY DSD^‡‡^ |  | Val |  | Met | Gln^¶^(5) | – | – | – | – | Val | – | – | His | Pro^¶^ | Asn | – | Leu | – | – | Thr^¶^ | – | – |
| gnomAD^§§^ |  | 1E-05 | 3E-05 | ND | 1E-06 | ND | 2E-06 | 6E-07 | 1E-05 | 3E-04 | ND | ND | 5E-05 | 2E-06 | 8E-05 | ND | ND | ND | ND | 7E-06 | 2E-04 | 4E-05 |
| ToMMo^¶¶^ |  | 8E-06 | ND | ND | 8E-06 | ND | 8E-06 | ND | 5E-04 | 8E-06 | ND | ND | ND | ND | 1E-03 | ND | ND | ND | ND | ND | 2E-05 | 8E-06 |
| Reference number in this table | [13] | [12] | [5] | [4][7] | [1] [2] [7] [8] [9] [12] [13] | [8] [13]  This report (2) | [13] | [10] | This report (1) | [3] [9] | [1] [13] | [11] | [13] | [4] | [12] | [2] [4] | [6] | [11] | [2] [3] [8]  This report (1) | [4] | [9] | [8] |
| ^†^Bold text indicates variants identified in this study. Numbers in parentheses indicate the number of patients.  Numbers in brackets refer to referencing studies in this table. | | | | | | | | | | | | | | | | | | | | | |  |
| ^‡^Ia, Ib, IV, V: RNA-binding motif, III, Va, VI: ATP-binding motif (Boneberg et al., 2019) | | | | | | | | | | | | | | | | | | | | | |  |
| ^§^Includes partially virilized 46,XY DSD [1] and gonadal dysgenesis | | | | | | | | | | | | | | | | | | | | | |  |
| ^¶^Includes patients with variants in *DHX37* and *NR5A1* [4] [5] [9], *DHX37* and *WWOX* [4], *DHX37* and *MAMLD1* [7], and *DHX37* and *SOX9* [this report] | | | | | | | | | | | | | | | | | | | | | |  |
| ^#^Includes embryonic testicular regression syndrome [2] | | | | | | | | | | | | | | | | | | | | | |  |
| ^††^Homozygous variant | | | | | | | | | | | | | | | | | | | | | |  |
| ^‡‡^Includes DSD with clinically unknown etiology, 46,XY DSD, gonadectomized, 46,XY DSD with primary amenorrhea | | | | | | | | | | | | | | | | | | | | | |  |
| ^§§^gnomAD, Genome Aggregation Database (https://gnomad.broadinstitute.org/) v4.1.0 | | | | | | | | | | | | | | | | | | | | | |  |
| ^¶¶^jMorp, Tohoku Medical Megabank Organization, Japanese Multi Omics Reference Panel (https://jmorp.megabank.tohoku.ac.jp/) 60KJPN AF | | | | | | | | | | | | | | | | | | | | | |  |
| ND: no determined | | | | | | | | | | | | | | | | | | | | | |  |
| [1] Buonocore et al., 2019; PMID: 31745530; [2] da Silva et al., 2019; PMID: 31287541; [3] Globa et al., 2022; PMID: 35432193; [4] Gomes et al., 2022; PMID: 35134971; [5] Kouri et al 2024; PMID: 38168586; [6] Kulkarni et al., 2023; PMID: 36617173; [7] Margiotti et al., 2024; PMID: 38962685; [8] McElreavey et al., 2020; PMID: 31337883; [9] Oliveira et al., 2023; PMID: 37240737; [10] Wan et al., 2023; PMID: 37065748; [11] Yang et al., 2023; PMID: 37717579; [12] Zhang et al., 2024; PMID: 37147882; [13] Zidoune et al., 2021; PMID: 34293745 | | | | | | | | | | | | | | | | | | | | | |  |

| **Table S2. Clinical characteristics of the six 46,XYDSD patients who have combined presence of heterozygous rare variant with *DHX37* and known 46,XYDSD causative genes.** | | | | | | | | | | | | | | | | | | | | | |
| --- | --- | --- | --- | --- | --- | --- | --- | --- | --- | --- | --- | --- | --- | --- | --- | --- | --- | --- | --- | --- | --- |
| Ref No. | Diagnosis (year or month) | *DHX37* variant  (ACMG^a^) | Other variant  (ACMG^a^) | Sex | EMS | Length of phallus | Testicular palpation | Urethral meatus | Scrotum/labia | Vagina | Gonads | Epididymis | Uterus | Fallopian tubes/ oviduct | Treatment |  | LH mIU/mL  [reference] |  | FSH mIU/mL  [reference] |  | T ng/dl  [reference] |
| 7 | PGD  (17 y) | Leu467Val  (VUS) | *NR5A1*  Met98Glyfs | Female | 0 | 3 cm | Not palpable | female type | Labia | NA | Abdominal  L: dysgenic testis (Sertoli cell present); R: streak gonad | NA | Absent | NA | NA |  | 13.9  [0.6–8.5] |  | 72.1  [0.5–9.5] |  | 70  [100–750] |
| 7 | PGD  (0.5 m) | Val999Met  (LB) | *NR5A1*  Ser4fs | Male | 6 | 2.2cm | Palpable | Peno /scrotum | Labia /scrotum | NA | LR: dysgenic testes | NA | Absent | NA | Preserving the gonad,  testosterone |  | 7.5 |  | 11.8 |  | 108  [131–871] |
| 8 | DSD  (0.3 y) | Ala494Pro  (VUS) | *NR5A1*  Gly26Val  (LP) | Male | NA | 3 cm | NA | Perineal | NA | NA | NA | NA | NA | NA | NA |  | NA |  | NA |  | 350 |
| 9 | PGD | Gly302Ser  (VUS) | *NR5A1*  Tyr404*  (LP) | Female | NA | NA | NA | NA | NA | NA | NA | NA | NA | NA | NA |  | NA |  | NA |  | NA |
| 10 | Amenorrhea  (15 y) | Arg308Gln  (P) | *MAMLD1*  Pro384Leu | Female | NA | 2.5 cm | Not palpable | NA | Labia | NA | NA | NA | Absent | NA | NA |  | NA |  | NA |  | NA |
| 8 | DSD  (12.9 y) | Ala737Thr  (VUS) | *WWOX* Tyr85Asp (VUS) | Male | NA | 6 cm | Not palpable | Perineal | NA | NA | Cryptorchidism | NA | NA | NA | NA |  | 3.3 |  | 7.0 |  | 619 |
| ^a^ Described as stated in the previous reports. P: pathogenic; LP: likely pathogenic; VUS: Variant of uncertain significance; LB: likely benign. | | | | | | | | | | | | | | | | | | | | | |
| Abbreviations: NA, no data available; PGD, partial gonadal dysgenesis; EMS, external masculinization score; LR, left and right; LH, luteinizing hormone; FSH, follicle-stimulating hormone; T, testosterone. | | | | | | | | | | | | | | | | | | | | | |
